# Resveratrol Induces Autophagy and Apoptosis in Non-Small-Cell Lung Cancer Cells by Activating the NGFR-AMPK-mTOR Pathway

**DOI:** 10.3390/nu14122413

**Published:** 2022-06-10

**Authors:** Jiaqiao Li, Yameng Fan, Yan Zhang, Yamei Liu, Yan Yu, Mao Ma

**Affiliations:** 1 School of Public Health, Xi’an Jiaotong University, Xi’an 710061, China; ljq0321@stu.xjtu.edu.cn (J.L.); fym0401@stu.xjtu.edu.cn (Y.F.); zhangyan_@stu.xjtu.edu.cn (Y.Z.); yameiliu170706@stu.xjt.edu.cn (Y.L.); 2 Physical Examination Department, The First Affiliated Hospital of Xi’an Jiaotong University, Xi’an 710061, China

**Keywords:** resveratrol, non-small-cell lung cancer, A549 cell line, autophagy, apoptosis, NGFR-AMPK-mTOR pathway

## Abstract

Resveratrol (RSV) has been reported to induce autophagy and apoptosis in non-small-cell lung cancer A549 cells, and the nerve growth factor receptor (NGFR) regulates autophagy and apoptosis in many other cells. However, the effect of NGFR on autophagy and apoptosis induced by RSV in A549 cells remains unclear. Here, we found that RSV reduced the cell survival rate in time- and concentration-dependent manners, activating autophagy and apoptosis. Lethal autophagy was triggered by RSV higher than 55 μM. The relationship between autophagy and apoptosis depended on the type of autophagy. Specifically, mutual promotion was observed between apoptosis and lethal autophagy. Conversely, cytoprotective autophagy facilitated apoptosis but was unaffected by apoptosis. RSV enhanced NGFR by increasing mRNA expression and prolonging the lifespan of NGFR mRNA and proteins. RSV antagonized the enhanced autophagy and apoptosis caused by NGFR knockdown. As the downstream pathway of NGFR, AMPK-mTOR played a positive role in RSV-induced autophagy and apoptosis. Overall, RSV-induced autophagy and apoptosis in A549 cells are regulated by the NGFR-AMPK-mTOR signaling pathway.

## 1. Introduction

Lung cancer has a substantial worldwide burden, and it has the highest mortality rate among solid tumors. More than 1.8 million people died from lung cancer worldwide in 2020, of which 85% were related to non-small-cell lung cancer (NSCLC) [1,2]. Due to the severe side effects and resistance to radiotherapy and chemotherapy, restricted patients and histological subtypes, and the emergence of secondary clones of immunotherapy and targeted therapy, the overall cure and survival rates of NSCLC remain low [2]. Therefore, novel drugs are greatly needed to improve the outcomes of NSCLC.

Resveratrol (3,5,4′-trihydroxystilbene, RSV), a natural non-flavone polyphenol compound, is a stilbene derived from *Polygonum cuspidatum*, *Cassia tora Linn*, and *Vitis vinifera* [3,4]. RSV attracts researchers’ attention due to its anticancer potential. RSV has been employed as an alternative drug to prevent and treat different types of cancer, such as breast, blood, kidney, liver, bladder, thyroid, prostate, lung, and skin cancer [3]. Numerous in vitro and in vivo studies have reported that RSV worked as a chemopreventive agent by producing proapoptotic, antiproliferation, and anti-inflammation effects [5]. In the last years, a number of studies have verified the anticancer effects of RSV on NSCLC, including the inhibition of cancer cell proliferation and tumor growth, cell cycle arrest restraint, ROS production enhancement, metastasis depression, and induction of cell apoptosis and autophagy [6]. Among these functions, apoptosis, termed type I programmed death, is a cellular death process regulated by multiple genes to maintain the homeostasis of the internal environment. It is accompanied by cellular shrinking, condensation, and margination of the chromatin, ruffling of the plasma membrane, and the appearance of apoptotic bodies [7]. As type II programmed cell death, autophagy (hereafter discussed as macroautophagy) is a cellular stress process to maintain intracellular homeostasis and integrity by packaging organelles and cytoplasmic macromolecules within double-membrane vesicles (autophagosomes). The vesicles are fused with lysosomes to form autolysosomes, in which autophagic cargoes are degraded [8]. Notably, autophagy induced by various types of stresses plays different roles in the fate of cells. In some circumstances, autophagy protects cells from death by removing protein aggregates and harmful organelles and regulating the cell cycle. In other situations, massive autophagy destroys most cytosol and organelles and triggers apoptosis or necrosis, thus resulting in a cellular suicide [9]. Almost all studies have explored one side of autophagy, either its promotion effect or inhibition effect on the survival of cells [10,11].

Considering the aim to simultaneously promote the survival of normal cells and inhibit survival of NSCLC cells by using RSV to promote autophagy, further studies are needed to clarify whether the role of autophagy induced by RSV is related to the concentration of RSV. The relationship between autophagy and apoptosis is complex due to the sharing of signaling pathways and the crosstalk between signal molecules. For instance, p53 and the mammalian target of rapamycin (mTOR) signal transduction pathway are known to regulate autophagy and apoptosis in NSCLC [12]. Moreover, the apoptotic roles of autophagy-related proteins and autophagic functions of caspases further confound the two processes [12]. The autophagy-apoptosis crosstalk has broad implications on cancer [8]. Unfortunately, few studies have mentioned the interplay between autophagy and apoptosis aroused by RSV in NSCLC [10,11]. The correlation has not been elucidated due to the dual role of autophagy.

The nerve growth factor receptor (NGFR, also known as p75NTR or CD271) is a member of the tumor necrosis factor receptor family. NGFR plays a role in various biological processes, such as cell proliferation, differentiation and death, scar formation, energy expenditure, and hypoxic response [13]. Further studies have revealed that NGFR regulates cell growth, invasion, metastasis, autophagy, and apoptosis in many tumors [13,14,15]. For example, RSV treatment reduced the histone methyltransferase EZH2 significantly, further led to reactivation of neuroblastoma tumor suppressor genes NGFR, and then triggered cellular apoptosis in *N*-2a cells [16]. However, the role of NGFR in lung cancer has not been clarified. As a cellular energy sensor, AMP-activated protein kinase (AMPK) coordinates metabolic pathways, copes with stress, and maintains cellular homeostasis [17]. The effects of AMPK on autophagy have attracted much attention because autophagy also maintains intracellular homeostasis [18]. A recent study implied that nerve growth factor targets NGFR and increase AMPK and mTOR phosphorylation to activate autophagy in Schwann cells [19]. The AMPK-mTOR pathway plays a positive or negative role in apoptosis [20,21]. (Z)3,4,5,4′-trans-tetramethoxystilbene, a new analogue of RSV, induces caspase-independent apoptosis and autophagy by activating AMPK and suppressing mTOR in NSCLC [22]. Here, we aimed to clarify whether the NGFR-AMPK-mTOR axis participates in RSV-induced autophagy and apoptosis. The present study investigates the molecular mechanism of RSV-induced autophagy and apoptosis in NSCLC A549 cells. First, we determined the RSV concentration threshold for promoting the lethal effect of autophagy using MTT assay and autophagy inhibitor 3-MA. Then, based on the RSV concentration threshold, we induced protective autophagy and lethal autophagy with different concentrations of RSV in A549 cells. We explored the relationship between RSV-induced autophagy and apoptosis by flow cytometry. Finally, considering the complex crosstalk between autophagy and apoptosis, we probed the impacts of the NGFR-AMPK-mTOR signaling pathway on autophagy and apoptosis in A549 cells. This study aimed to support the development of a new targeted therapy for NSCLC by revealing the detailed mechanism of RSV-induced autophagy and apoptosis.

## 2. Methods

### 2.1. Cell Culture and Treatment

Lung adenocarcinoma is the largest subtype of NSCLC, with approximately 40–50% of all cases [23]. Therefore, we selected human lung adenocarcinoma A549 cells as the object of the study. A549 cell line was purchased from the National Infrastructure of Cell Line Resource (Beijing, China) and cultured in Roswell Park Memorial Institute (RPMI)-1640 medium (Hyclone, Logan, UT, USA) with 10% (*v*/*v*) fetal bovine serum (FBS, Biological Industries, Kibbutz Beit Haemek, Israel) and 1% (*v*/*v*) penicillin-streptomycin liquid (Solarbio, Beijing, China). Human bronchial epithelial BEAS-2B cell line was used as a normal cell line; it was provided by Associate Professor Jianhua Yi (Xi’an Jiaotong University, Xi’an, China) and cultured in Dulbecco modified Eagle medium (DMEM, HyClone, Logan, UT, USA) with 10% (*v*/*v*) FBS and 1% (*v*/*v*) penicillin–streptomycin liquid. All cells were grown in a humidified incubator at 37 °C and 5% CO_2_.

RSV, autophagy inhibitor 3-MA, and apoptosis inhibitor Z-VAD-FMK were purchased from Sigma-Aldrich (St. Louis, MO, USA). Compound C (Cpd. C, AMPK inhibitor) was purchased from Aladdin (Shanghai, China). RSV was dissolved in DMSO (MP Biomedicals, Irvine, CA, USA) at a concentration of 200 mM as the stock solution. The stock solution was diluted in medium to prepare the working solutions before each experiment. Before RSV treatment, 3-MA (5 mM) and Z-VAD-FMK (30 μM) were applied to pretreat cells for 1 and 0.5 h, respectively.

### 2.2. Cell Viability Analysis

Cell viability was assessed via MTT assay. Cells were seeded into a 96-well plate with 6000 cells per well. At the end of the intervention period, the cell medium was removed, and 20 μL of 5 mg/mL MTT (MP Biomedicals) was added to each well. After incubating for 4 h at 37 °C in the dark, 150 μL of DMSO (MP Biomedicals) was added, and the optical density was measured at 490 nm with a microplate reader [24].

### 2.3. Monodansylcadaverine (MDC) Staining

Immediately after RSV treatment, the cell culture medium was removed, and the culture plate was washed with PBS twice. MDC (50 μM, Sigma-Aldrich) was applied to stain the cells for 15 min at 37 °C in the dark [25]. Cells were photographed under a fluorescence microscope. Mature autophagy vacuoles were dyed by MDC.

### 2.4. Acridine Orange (AO) Staining

AO (Sigma-Aldrich) fluorescence intensity was determined by flow cytometry to evaluate autophagy. Briefly, cells were gathered with EDTA-free trypsin, rinsed with PBS, and incubated in 1 μg/mL AO for 15 min at 37 °C in the dark [26].

### 2.5. Transmission Electron Microscopy

Cells were harvested after specific treatment and washed with PBS twice. Cell clusters were fixed in 2.5% glutaraldehyde (in 0.1 M phosphate buffer with 4% paraformaldehyde) at 4 °C for 4 h and in 1% osmic anhydride (in 0.1 M phosphate buffer) at 4 °C for 2 h. The specimens were dehydrated using gradient ethanol solutions (30% for 10 min, 50% for 10 min, 70% for 10 min, uranyl acetate in 70% ethanol for 2 h, 90% for 10 min twice, and 100% for 10 min thrice). The specimens were transferred to propylene oxide for 10 min, soaked and embedded with epoxy resin, and semi-sectioned at 1–2 μm. Subsequently, samples were dyed by methylthionine chloride, ultrathin sectioned at 50–70 nm, and stained with uranium acetate and lead citrate [25]. A transmission electron microscope was used to observe and photograph autophagosomes, autolysosomes, and apoptosomes.

### 2.6. Adenovirus mRFP-GFP-LC3 Fluorescence Assay

Cells were seeded in confocal dishes for 24 h and transfected with adenovirus mRFP-GFP-LC3 (MOI = 100, Hanbio Biotechnology, Shanghai, China) for 6 h before specific treatment. A confocal microscope visualized cells. Yellow puncta represent autophagosomes, while red puncta indicate autolysosomes, as green fluorescent protein is intolerant of the lysosomal acidic conditions. Autophagic flux was evaluated by the ratio of red puncta to yellow puncta [27].

### 2.7. Annexin V-FITC/PI Staining and TUNEL Staining

After specific treatment, cells were collected with EDTA-free trypsin, gently washed with PBS, and suspended in binding buffer (2 × 10^5^ cells/mL). Subsequently, an annexin V-FITC/propidium iodide (PI) detection kit (7 Sea Biotech, Shanghai, China) or terminal deoxynucleotidyltransferase-mediated dUTP nick end labeling (TUNEL)-FITC/PI detection kit (7 Sea Biotech) was utilized to double-stain the cells in accordance with the manufacturer’s instructions. The ratio of apoptotic cells was assessed via flow cytometry.

### 2.8. Cell Transfection and RNA Knockdown

Short interfering RNA (siRNA) transfection was carried out with GP-transfect Mate (Genepharma, Shanghai, China) according to the manufacturer’s instructions. Negative control siRNA was ordered from Genepharma, which targets 5′-UUCUCCGAACGUGUCACGUTT-3″. The sequence for NGFR siRNA was as follows: NGFR#1: 5′-CCUUCAAGAGGUGGAACAGTT-3′; NGFR#2: 5′-GCUACUACCAGGAUGAGACTT-3′. The sequence for AMPK siRNA was as follows: AMPK#1: 5′-GGAGAGCUAUUUGAUUAUATT-3′; AMPK#2: 5′-GCGUGUACGAAGGAAGAAUTT-3′.

### 2.9. mRNA Stability Analysis

Cells were pretreated with 80 μM RSV for 48 h and then incubated with 5 μg/mL actinomycin D (Sigma-Aldrich) for 0, 3, and 6 h to block global mRNA transcription [28]. Total RNA was extracted by TRIzol reagent and reversely transcribed to synthesize cDNA. The mRNA levels were determined by qRT-PCR.

### 2.10. RNA Extraction and Quantitative Real-Time Polymerase Chain Reaction (qRT-PCR)

Total RNA in cells was extracted with TRIzol reagent (Invitrogen, Carlsbad, CA, USA). After quantifying and testing the purity of the extracted RNA by NanoDrop (Thermo Fisher Scientific, Carlsbad, CA, USA), 500 μL of total RNA was reversely transcribed to synthesize cDNA using PrimerScript RT Master Mix (Takara, Dalian, China). QRT-PCR was carried out with TB Green Premix Ex Taq II (Takara) in a LightCycler 96 PCR system (Roche, Basel, Switzerland), according to the manufacturer’s guidelines. The relative expression of each gene was calculated by the 2^−ΔΔCt^ method normalized to ACTB. The primers can be found in Table 1.

### 2.11. Protein Stability Analysis

Cells were pretreated with 80 μM RSV for 48 h and then incubated with 40 μg/mL cycloheximide (Sigma-Aldrich) for 0, 4, 8, and 12 h [28]. Protein was harvested by RIPA lysis buffer and detected by Western blot.

### 2.12. Western Blot Analysis

Total protein was isolated by RIPA lysis buffer (Beyotime, Shanghai, China) with 1% PMSF (Beyotime) and quantified by BCA Protein Assay Kit (Beyotime). Thirty micrograms of protein were separated by SDS-PAGE (Beyotime) and transferred to polyvinylidene difluoride (PVDF, Millipore, Billerica, MA, USA) membranes. The membranes were blocked with QuickBlock Blocking Buffer (Beyotime) for 30 min at room temperature before incubating with primary antibodies at 4 °C overnight. Subsequently, the membranes were incubated with secondary antibodies conjugated with horseradish peroxidase (HRP, Proteintech, Rosemont, IL, USA) for 1 h at room temperature. Protein blots were scanned and imaged by enhanced chemiluminescence (Luminata Forte, Millipore) [21]. ACTB was used to normalize the relative expression of proteins. The primary antibodies used were BECLIN 1 (#3495, CST, Cell Signaling Technology, Danvers, MA, USA), LC3B (#2775, CST), BAX (#ab32503, Abcam, Cambridge, UK), BCL-2 (#ab182858, Abcam), NGFR (#ab52987, Abcam), p-AMPK (#2535, CST), AMPK (#5831, CST), p-mTOR (#5536, CST), mTOR (#2983, CST), and ACTB (#66009-1-lg, Proteintech, Rosemont, IL, USA).

### 2.13. Statistical Analysis

Experiment data were expressed as the mean ± standard deviation and were statistically analyzed by one-way ANOVA with Tukey’s test or Student’s *t*-test with SPSS 18.0 (IBM, Armonk, NY, USA). A *p* value < 0.05 was considered statistically significant. Each experiment was repeated at least three times independently.

## 3. Results

### 3.1. The Effect of RSV on Cell Viability in A549 and BEAS-2B Cells

**Figure 1:**
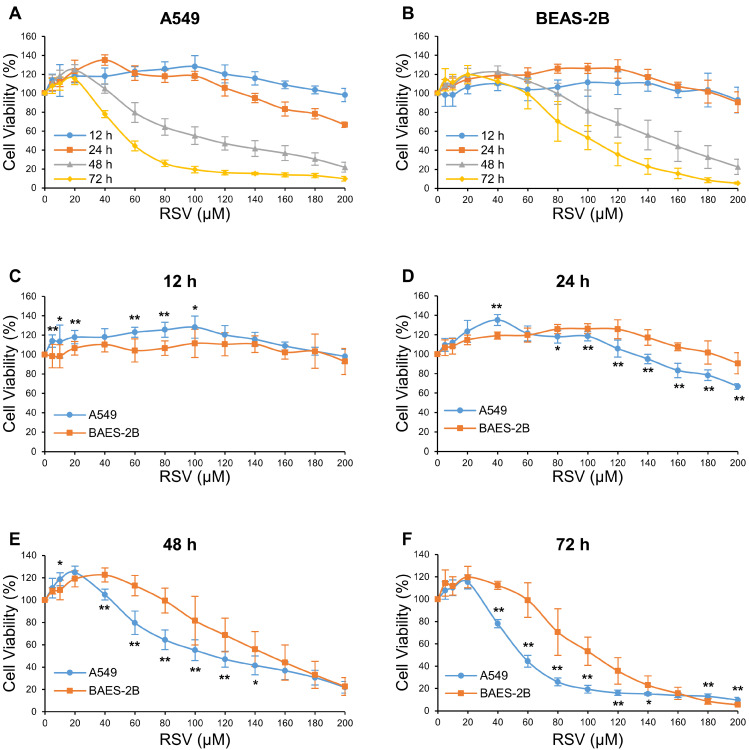
The effect of resveratrol (RSV) on cell viability in A549 and BEAS-2B cells. A549 cells (**A**) and BEAS-2B cells (**B**) were treated with RSV (0–200 μM) for 12 h (**C**), 24 h (**D**), 48 h (**E**), and 72 h (**F**). Cell viability was determined by the MTT assay. The data are presented as mean ± SD from three independent experiments. * *p* < 0.05 and ** *p* < 0.01 compared with BEAS-2B group.

The impacts of RSV on the cell viability of human lung adenocarcinoma A549 cells and human bronchial epithelial BEAS-2B cells were evaluated by MTT assay. Two cell lines were treated with corresponding RSV concentrations (0, 5, 10, 20, 40, 60, 80, 100, 120, 140, 160, 180, and 200 μM) and duration (12, 24, 48, and 72 h) to select the appropriate treatment conditions. The results showed that lower concentration (5–20 μM) or shorter duration (12 h) of RSV treatment promoted cell viability of both A549 and BEAS-2B cells, compared with the control group. The decrease in cell survival rate was concentration- and time-dependent, as RSV concentration increased and duration extended (Figure 1). We selected a duration and concentration range in which the cell viability of A549 cells was inhibited while that of BEAS-2B cells was improved. Only a high concentration (≥140 μM) of RSV inhibited A549 cell viability in the 24 h group (*p* < 0.05). Treating with RSV for 72 h damaged A549 cell seriously, while 40 μM and 60 μM RSV reduced cell viability to 78 and 44%, respectively (*p* < 0.05). These concentrations might induce other types of programmed cell death instead of autophagy or apoptosis [12]. The survival rate of BEAS-2B cells was significantly higher than those of A549 cells after treatment with RSV at 40–140 μM for 48 h (*p* < 0.05). Therefore, the duration of 48 h and RSV concentrations lower than 150 μM was used in the next experiment.

### 3.2. Determination of RSV Concentration Threshold for Inhibiting the Survival Effect of Autophagy in A549 and BEAS-2B Cells

**Figure 2:**
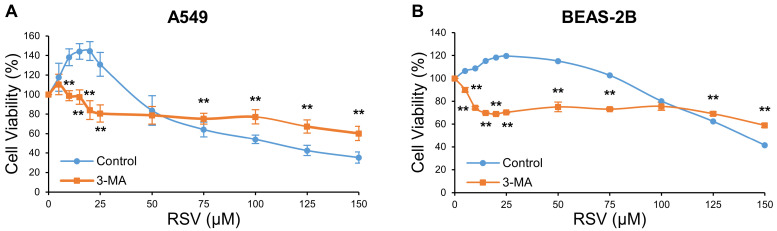
Determination of resveratrol (RSV) concentration threshold for inducing lethal autophagy in A549 and BEAS-2B cells. A549 cells (**A**) and BEAS-2B cells (**B**) were treated with RSV (0–150 μM) in the presence or absence of 5 mM 3-MA for 48 h. Cell viability was determined by the MTT assay. The data are presented as mean ± SD from three independent experiments. ** *p* < 0.01 compared with the control group.

Autophagy plays dual roles in cell viability, it can be protective or lethal [9]. To explore whether the function of RSV-induced autophagy was related to RSV concentration, MTT assay and autophagy inhibitor 3-MA were utilized in the following experiments. At low RSV concentration, the survival rate was suppressed, while in high concentration, the survival rate was elevated when autophagy was inhibited, compared with the corresponding control group (Figure 2). The RSV concentration thresholds were 55 and 108 μM in A549 and BEAS-2B cells. Thus, RSV concentrations from 55 to 108 μM activated lethal autophagy in A549 cells and protective autophagy in BEAS-2B cells. Based on these results, we inferred an RSV concentration of 81.5 μM (the average value of 55 and 108 μM) and 28.5 μM (linear decreasing term of 81.5 and 55 μM) could be used to induce A549 cells death-promoting autophagy and survival-promoting autophagy during a 48 h treatment, respectively. In practice, we finally selected the concentrations of 80 and 30 μM in the following experiments.

### 3.3. RSV Induces Autophagy and Apoptosis in A549 Cells

**Figure 3:**
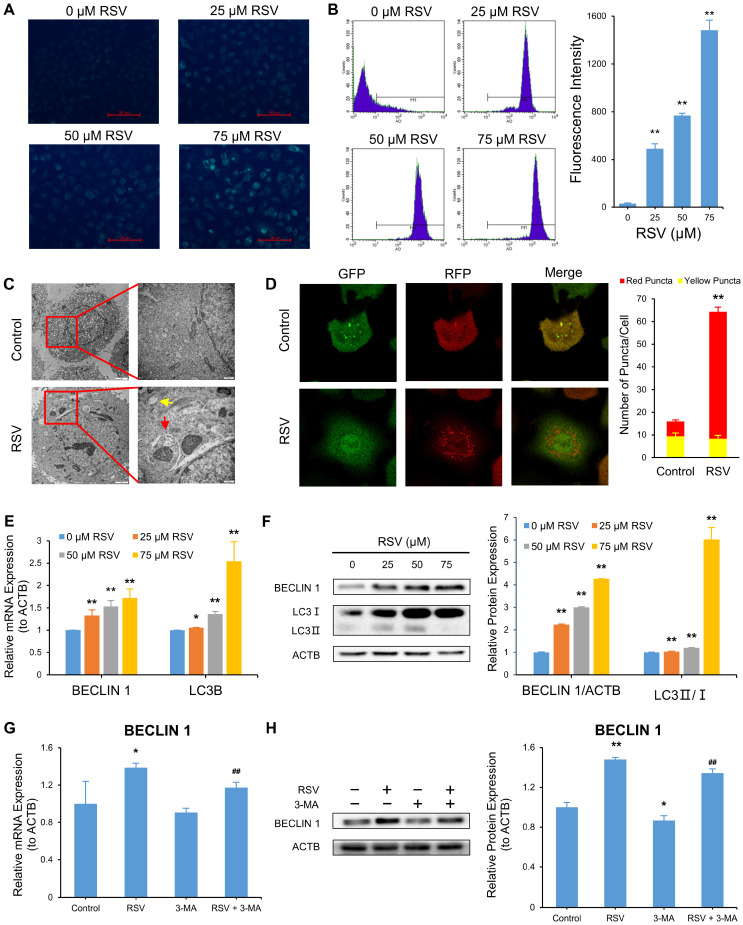
Resveratrol (RSV) promotes autophagy in A549 cells. Cells were treated with various concentrations of RSV for 48 h. (**A**) MDC (50 μM) fluorescent staining was used to mark autophagosomes; (**B**) AO (1 μg/mL) staining of autophagic vesicles. Fluorescence intensity was determined by flow cytometry (APC); (**C**) Autophagosomes (marked by red arrow) and autolysosomes (marked by yellow arrow) observed under TEM; (**D**) Cells were infected by adenovirus mRFP-GFP-LC3. Autophagosomes (yellow puncta) and autolysosomes (red puncta) were observed under confocal microscope; (**E**) Transcriptional expression of BECLIN 1 and LC3B as determined by qRT-PCR. ACTB was used as an internal control; (**F**) Protein expression of BECLIN 1, LC3 I, and LC3 II as determined by Western blot. ACTB was used as an internal control; (**G**) QRT-PCR analysis of mRNA level of BECLIN 1 in the cells treated with or without 80 μM RSV or 5 mM 3-MA. ACTB was used as an internal control; (**H**) Western blot assay of protein level of BECLIN 1 in the cells treated with or without 80 μM RSV or 5 mM 3-MA. ACTB was used as an internal control. The data are presented as the mean ± SD of triplicate tests. * *p* < 0.05 and ** *p* < 0.01 compared with the control group. ## *p* < 0.01 compared with 80 μM RSV group.

We applied MDC and AO staining to detect autophagy and transmission electron microscopy to observe autophagosomes and autolysosomes. Adenovirus mRFP-GFP-LC3 transfecting assay, qRT-PCR, and Western blot were also employed. MDC, an acidophilic fluorescent dye, marks autophagosomes precisely [29]. Figure 3A shows the green fluorescence signals were elevated as RSV concentration increased. Next, AO was used to stain the nucleus and cytoplasm bright green, and autophagic vesicles appeared bright red [30]. Flow cytometry was used to monitor autophagic vesicles. RSV treatment elevated the fluorescence intensity in a dose-dependent manner (Figure 3B). Then, we observed autophagosomes and autolysosomes in RSV-treated A549 cells, which were rare in the control group (Figure 3C). To clarify the changes in autophagy molecular markers, we first monitored autophagy flux using adenovirus mRFP-GFP-LC3. The ratio of red puncta (autolysosomes) to yellow puncta (autophagosomes) increased in cells treated with RSV (Figure 3D), indicating a more substantial autophagy flux. Subsequently, we applied qRT-PCR and Western blot to determine the mRNA and protein level, respectively, of autophagy biomarkers BECLIN 1 and LC3. Compared with the control group, RSV treatment significantly augmented BECLIN 1 and LC3B levels and the ratio of LC3 II: LC3 I in a dose-dependent manner (Figure 3E,F). Autophagy inhibitor 3-MA reversed the enhanced BECLIN 1 and LC3B levels and the LC3 II: LC3 I ratio induced by RSV (Figure 3G,H). We evaluated autophagy at the microstructure, ultrastructure, transcriptional, and translational levels, and the results indicate RSV-activated autophagy in a dose-dependent manner in A549 cells.

**Figure 4:**
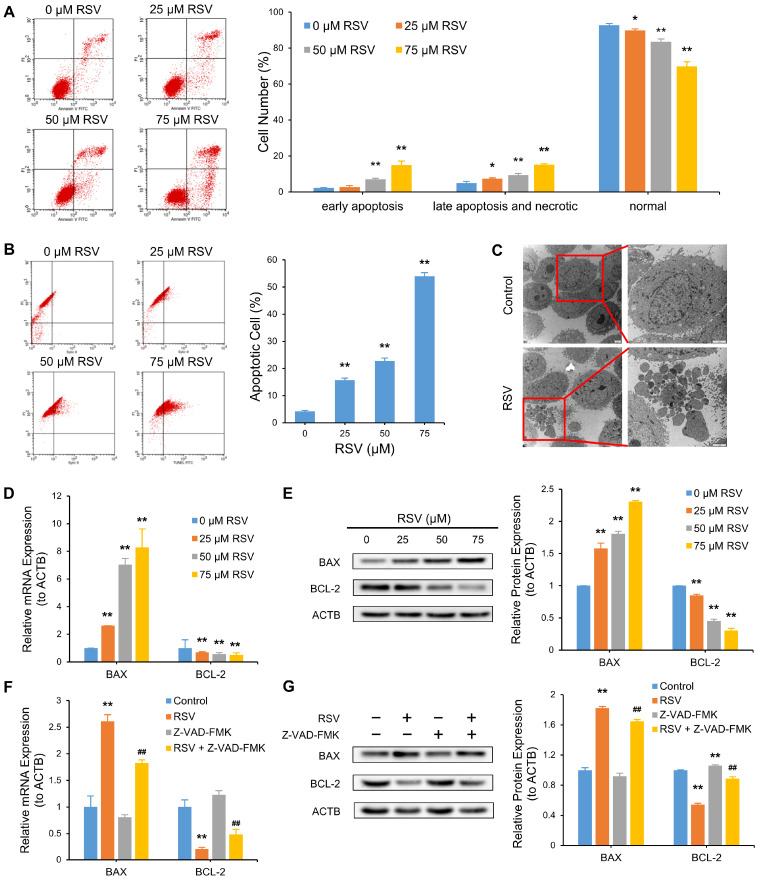
Resveratrol (RSV) induces apoptosis in A549 cells. Cells were treated with various concentrations of RSV for 48 h. (**A**) Annexin V-FITC/PI staining was used to detect the proportion of early apoptotic cells, late apoptotic and necrotic cells, and normal cells; (**B**) TUNEL-FITC/PI staining was used to detect the percentage of apoptosis cells; (**C**) Apoptosome observed under TEM; (**D**) Transcriptional expression of BAX and BCL-2 as determined by qRT-PCR. ACTB was used as an internal control; (**E**) Protein expression of BAX and BCL-2 as determined by Western blot. ACTB was used as an internal control; (**F**) QRT-PCR analysis of mRNA level of BAX and BCL-2 in the cells treated with or without 80 μM RSV or 30 μM Z-VAD-FMK. ACTB was used as an internal control; (**G**) Western blot assay of protein levels of BAX and BCL-2 in the cells treated with or without 80 μM RSV or 30 μM Z-VAD-FMK. ACTB was used as an internal control. The data are presented as the mean ± SD of triplicate tests. * *p* < 0.05 and ** *p* < 0.01 compared with the control group. ## *p* < 0.01 compared with 80 μM RSV group.

To verify whether the RSV-induced attenuation of cell viability in A549 cells was related to apoptosis, we measured the apoptotic cell ratio through annexin V-FITC/PI staining and TUNEL-FITC/PI staining with flow cytometry, separately. The data demonstrate the proportions of early apoptosis cells (right lower quadrant), late apoptosis, and necrotic cells (right upper quadrant) increased from 2 to 15% and from 5 to 15%, respectively, while that of normal cells (left lower quadrant) decreased from 93 to 70% in a dose-dependent manner (*p* < 0.01) (Figure 4A). Consistently, the ratio of TUNEL-positive cells (right upper quadrant) to PI-positive cells (left and right upper quadrant) was also elevated (from 4 to 54%, *p* < 0.01) (Figure 4B). We observed apoptosomes in RSV-treated A549 cells under a transmission electron microscope, absent in the control group (Figure 4C). QRT-PCR and Western blot detected apoptosis biomarkers, BAX and BCL-2, at the transcriptional and translational levels, respectively. BAX was elevated as RSV concentration increased, while BCL-2 was suppressed (Figure 4D,E). Apoptosis inhibitor Z-VAD-FMK reversed the RSV-induced changes in BAX and BCL-2 to some degree (Figure 4F,G). The data indicate RSV induces apoptosis in A549 cells.

### 3.4. Relationship between RSV Induced-Autophagy and Apoptosis

**Figure 5:**
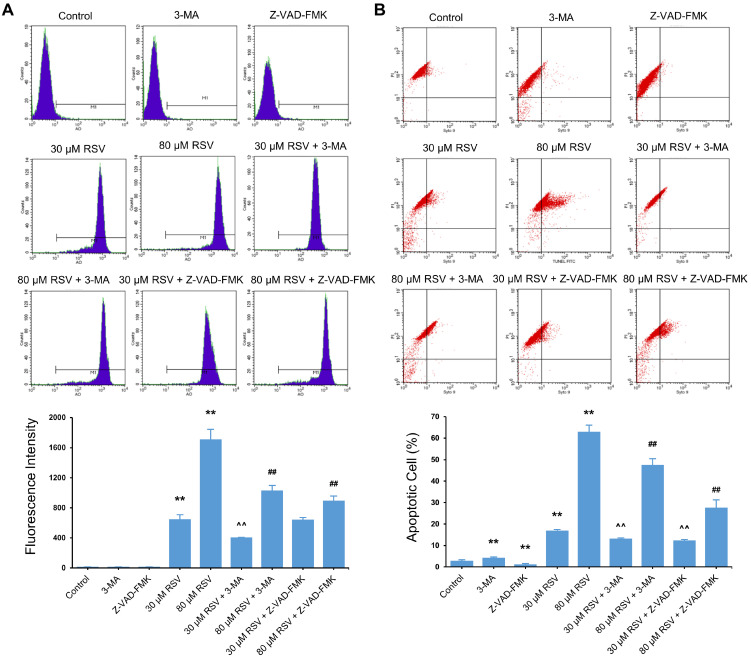
Functional relationship between autophagy and apoptosis induced by resveratrol (RSV) in A549 cells. Cells were incubated with RSV (30 or 80 μM) alone or in combination with 5 mM 3-MA or 30 μM Z-VAD-FMK for 48 h. (**A**) AO (1 μg/mL) staining of autophagic vesicles. Fluorescence intensity was determined by flow cytometry (APC); (**B**) TUNEL-FITC/PI staining was used to detect the percentage of apoptosis cells. The data are presented as mean ± SD from three independent experiments. ** *p* < 0.01 compared with the control group. ^^ *p* < 0.01 compared with 30 μM RSV group. ## *p* < 0.01 compared with 80 μM RSV group.

To clarify the relationship between protective and lethal autophagy and apoptosis, we induced protective and lethal autophagy with 30 μM and 80 μM RSV in A549 cells. The apoptosis rate and autophagy level were measured by TUNEL-FITC/PI staining and AO staining, respectively, in combination with flow cytometry. Cell apoptosis was suppressed when either cytoprotective autophagy or lethal autophagy was inhibited (Figure 5A). Conversely, inhibiting apoptosis did not affect cytoprotective autophagy but attenuated lethal autophagy (Figure 5B).

### 3.5. RSV Increases NGFR by Enhancing the Stability of mRNA and Protein

**Figure 6:**
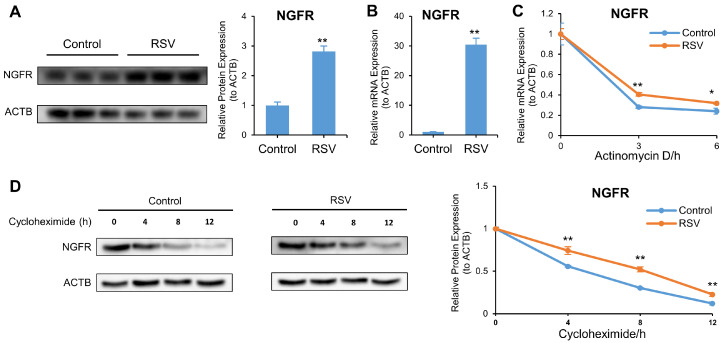
Resveratrol (RSV) increases NGFR by enhancing the stability of mRNA and protein. Cells were treated with or without 80 μM RSV for 48 h. (**A**) Protein expression of NGFR as determined by Western blot. ACTB was used as an internal control; (**B**) Transcriptional expression of NGFR as determined by qRT-PCR. ACTB was used as an internal control; (**C**) Lifespan of NGFR mRNA as determined by qRT-PCR in cells incubated with actinomycin D for 0, 3, or 6 h after RSV treatment. ACTB was used as an internal control; (**D**) Lifespan of NGFR protein as determined by Western blot in cells incubated with cycloheximide for 0, 4, 8, or 12 h after RSV treatment. ACTB was used as an internal control. The data are presented as mean ± SD from three independent experiments. * *p* < 0.05 and ** *p* < 0.01 compared with the control group.

We examined whether RSV affected autophagy and apoptosis through the NGFR pathway in A549 cells. Western blot results show RSV upregulated NGFR protein by 176%, compared with the control (*p* < 0.01) (Figure 6A). To understand the mechanism behind this result, we determined NGFR mRNA expression by qRT-PCR, mRNA stability by actinomycin D, and protein stability by cycloheximide. RSV elevated NGFR mRNA and retarded NGFR mRNA and protein degradation after actinomycin D and cycloheximide treatment. This implies RSV increased NGFR protein expression by increasing mRNA expression and prolonging the lifespan of mRNA and protein (Figure 6B–D).

### 3.6. RSV Induces Autophagy and Apoptosis by Targeting NGFR

**Figure 7:**
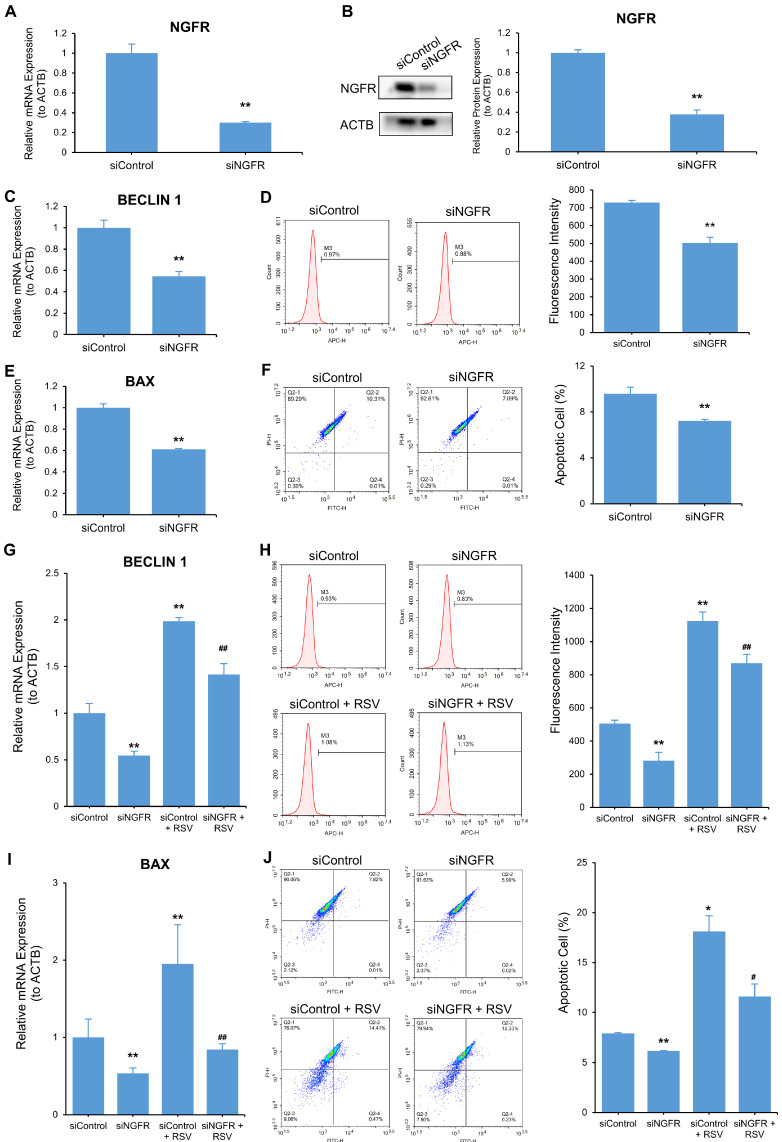
Resveratrol (RSV) induces autophagy and apoptosis by targeting NGFR. Cells were transfected with siControl or siNGFR in the presence or absence of 80 μM RSV for 48 h. (**A**) Transcriptional expression of NGFR as determined by qRT-PCR. ACTB was used as an internal control; (**B**) Protein expression of NGFR as determined by Western blot. ACTB was used as an internal control; (**C**) QRT-PCR analysis of BECLIN 1 mRNA. ACTB was used as an internal control; (**D**) AO (1 μg/mL) staining of autophagic vesicles. Fluorescence intensity was determined by flow cytometry (APC); (**E**) Transcriptional expression of BAX as determined by qRT-PCR. ACTB was used as an internal control; (**F**) TUNEL-FITC/PI staining was used to detect the percentage of apoptotic cells; (**G**) QRT-PCR analysis of BECLIN 1 mRNA. ACTB was used as an internal control; (**H**) AO (1 μg/mL) staining of autophagic vesicles. Fluorescence intensity was determined by flow cytometry (APC); (**I**) Transcriptional expression of BAX as determined by qRT-PCR. ACTB was used as an internal control; (**J**) TUNEL-FITC/PI staining was used to detect the percentage of apoptotic cells. The data are presented as mean ± SD from three independent experiments. * *p* < 0.05 and ** *p* < 0.01 compared with the control group. # *p * < 0.05 and ## *p* < 0.01 compared with siNGFR group.

To validate whether NGFR influenced RSV-induced autophagy and apoptosis, we knocked down NGFR using siRNA in A549 cells. The qRT-PCR and Western blot results validated the knockdown effectiveness (Figure 7A,B and Figure S1A,B). NGFR knockdown activated autophagy and apoptosis in A549 cells. Specifically, knockdown of NGFR increased autophagy marker BECLIN 1 mRNA (Figure 7C). A549 cells were stained with AO, and the fluorescence intensity was determined by flow cytometry. Cells transfected with siNGFR exhibited lower fluorescence intensity than the siControl group, implying weaker autophagy after NGFR knockdown (Figure 7D and Figure S1C). NGFR knockdown elevated the mRNA of apoptosis maker BAX (Figure 7E). TUNEL-FITC/PI double-staining results show the apoptosis rate decreased in siNGFR-transfected cells by 75%, compared with siControl-transfected cells (*p* < 0.01) (Figure 7F and Figure S1D).

We probed whether RSV could reverse the effect of NGFR knockdown on cell autophagy and apoptosis by qRT-PCR and AO staining. RSV reversed the impact of NGFR knockdown on the mRNA of autophagy marker BECLIN 1 (Figure 7G). After staining with AO, the fluorescence intensity of RSV-treated NGFR knockdown cells increased by 121%, compared with unhandled NGFR knockdown cells (*p* < 0.01) (Figure 7H). We used qRT-PCR and TUNEL-FITC/PI double-staining to evaluate cell apoptosis. RSV reversed the decrease in mRNA of apoptosis marker BAX induced by NGFR knockdown (Figure 7I). The rate of apoptotic cells was augmented in RSV-treated NGFR knockdown cells by 188%, compared with the unhandled NGFR knockdown cells (*p* < 0.05) (Figure 7J). RSV enhanced the inhibition of autophagy and apoptosis by NGFR knockdown in A549 cells.

### 3.7. RSV Stimulates NGFR Downstream Pathway AMPK-mTOR

**Figure 8:**
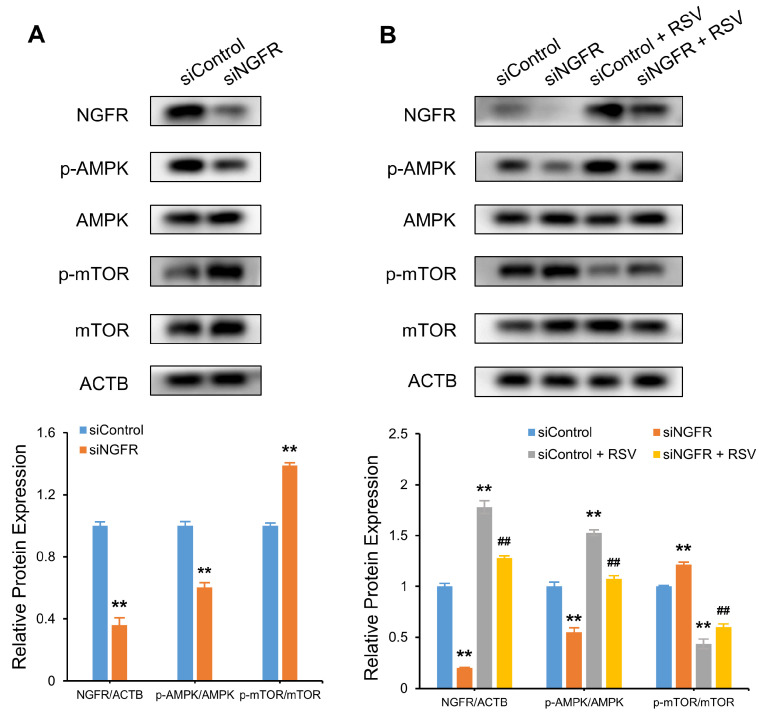
Resveratrol (RSV) stimulates the NGFR downstream pathway AMPK-mTOR. (**A**) Protein expression of p-AMPK, AMPK, p-mTOR, mTOR, and ACTB as determined by Western blot in cells with or without NGFR knockdown; (**B**) Protein expression of p-AMPK, AMPK, p-mTOR, mTOR, and ACTB as determined by Western blot in cells with or without NGFR knockdown, in the presence or absence of 80 μM RSV for 48 h. The data are presented as the mean ± SD of triplicate tests. ** *p* < 0.01 compared with the control group. ## *p* < 0.01 compared with siNGFR group.

The AMPK-mTOR signaling pathway is the downstream pathway of NGFR in Schwann cells, and it modulates autophagy at the early stage of peripheral nerve injury [19]. We explored whether NGFR stimulated the AMPK-mTOR pathway in A549 cells by phosphorylation. P-AMPK, AMPK, p-mTOR, and mTOR levels in siControl- or siNGFR-transfected A549 cells were determined by Western blot. Knockdown of NGFR suppressed p-AMPK by 80% and elevated p-mTOR by 227% (*p* < 0.01) (Figure 8A). RSV reversed the effect of NGFR knockdown on the AMPK-mTOR axis, as evidenced by the increase in p-AMPK by 134% and decrease in p-mTOR by 68% (*p* < 0.01) (Figure 8B). The results indicate RSV stimulated the NGFR downstream pathway, AMPK-mTOR.

### 3.8. Inhibition of AMPK Suppresses RSV-Mediated Autophagy and Apoptosis

**Figure 9:**
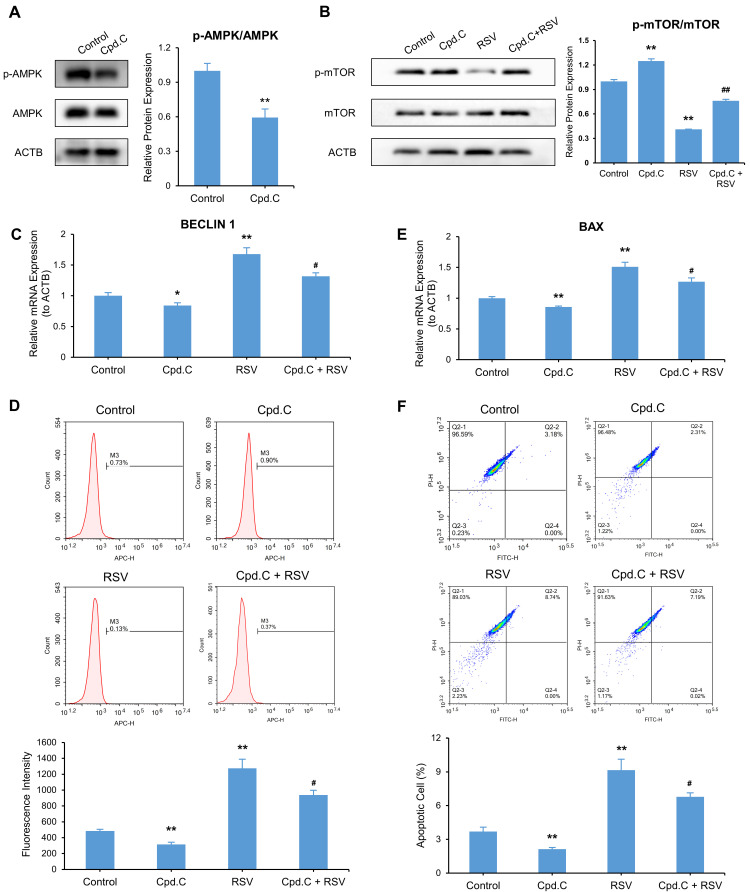
Inhibition of AMPK suppresses resveratrol (RSV)-mediated autophagy and apoptosis. Cells were treated with or without Cpd. C, in the presence or absence of 80 μM RSV for 48 h. (**A**) Protein expression of p-AMPK, AMPK, and ACTB as determined by Western blot; (**B**) Protein expression of p-mTOR, mTOR, and ACTB as determined by Western blot; (**C**) Transcriptional expression of BECLIN 1 as determined by qRT-PCR. ACTB was used as an internal control; (**D**) AO (1 μg/mL) staining of autophagic vesicles. Fluorescence intensity was determined by flow cytometry (APC); (**E**) QRT-PCR analysis of BAX mRNA. ACTB was used as an internal control; (**F**) TUNEL-FITC/PI staining was used to detect the percentage of apoptotic cells. The data are presented as mean ± SD from three independent experiments. * *p* < 0.05 and ** *p* < 0.01 compared with the control group. # *p* < 0.05 and ## *p* < 0.01 compared with RSV group.

To determine the effects of AMPK on autophagy and apoptosis in A549 cells, we knocked down AMPK by AMPK inhibitor Cpd. C and siRNAs separately. The knockdown effectiveness of Cpd. C was evaluated by Western blot and Cpd. C suppressed the p-AMPK/AMPK ratio by 59% (*p* < 0.01) (Figure 9A). The qRT-PCR and Western blot results confirmed the knockdown effected by siRNAs (Figure S2A,B). To validate the downstream pathway of AMPK, we measured p-mTOR and mTOR levels in Cpd. C-treated A549 cells, with or without RSV treatment. AMPK knockdown elevated the p-mTOR/mTOR ratio by 125% (*p* < 0.01). The results indicate mTOR is the downstream pathway of AMPK, and AMPK activated mTOR by phosphorylation. RSV antagonized the enhanced p-mTOR/mTOR ratio in AMPK knockdown cells by 61% (*p* < 0.01) (Figure 9B).

To clarify whether knockdown of AMPK inhibited RSV-induced autophagy and apoptosis, qRT-PCR and AO staining were carried out to determine autophagy. QRT-PCR and TUNEL-FITC/PI double-staining were used to determine the apoptosis rate. Knockdown of AMPK decreased RSV-enhanced BECLIN 1 mRNA expression (Figure 9C). After AO staining, the fluorescence intensity was suppressed in cells co-treated with Cpd. C and RSV, as well as in cells co-treated with siAMPK and RSV, compared with the cells treated with RSV alone (Figure 9D and Figure S2C). AMPK knockdown counteracted the effect of RSV on BAX (Figure 9E). The cell apoptosis rate of cells co-treated with Cpd. C and RSV decreased from 9.1 to 6.8%, compared with the cells treated with RSV alone (*p* < 0.05) (Figure 9F). Similar phenomena were observed in cells co-treated with siAMPK and RSV (Figure S2D). These results imply AMPK knockdown reversed RSV-induced autophagy and apoptosis in A549 cells.

## 4. Discussion

Using A549 cells, the positive effect of RSV on the activation of autophagy and apoptosis in NSCLC was investigated, and its molecular mechanisms were elucidated. The findings in the present study were: (1) The RSV concentration threshold to trigger lethal autophagy was 55 μM in A549 cells. Therefore, protective autophagy was induced by RSV lower than 55 μM, while lethal autophagy was induced by RSV higher than 55 μM. (2) The relationship between RSV-induced autophagy and apoptosis is dependent on the autophagy type. Lethal autophagy and apoptosis promoted each other, while cytoprotective autophagy facilitated apoptosis but was unaffected by apoptosis. (3) RSV-induced autophagy and apoptosis in A549 cells were likely regulated by the NGFR-AMPK-mTOR signaling pathway. Our study provides a novel mechanistic insight into the effect of RSV on enhancing autophagy and apoptosis of NSCLC A549 cells.

Our study confirmed RSV-induced autophagy and apoptosis in A549 cells at the microstructure, ultrastructure, transcriptional, and translational levels. Autophagosomes, autolysosomes, and apoptosomes were observed in RSV-treated A549 cells, which were rare or absent in the control group. In addition, autophagy flux was elevated in RSV-treated A549 cells, as evidenced by an increase in red puncta (representing autolysosomes) and a decrease in yellow puncta (representing autophagosomes). The results from qRT-PCR and Western blot analyses showed that RSV treatment significantly suppressed BCL-2 levels and augmented BECLIN 1, LC3B, and BAX levels and the ratio of LC3 II/LC3 I, in a dose-dependent manner. These results suggest RSV activated autophagy and apoptosis in a concentration-dependent way in A549 cells, and is in line with the findings of previous reports [10,31]. Autophagy and apoptosis are two forms of programmed death. Apoptosis promotes cell death, while autophagy has conflicting roles in cell viability [9]. Autophagy mainly protects cells from death as a mechanism to adapt to stress [32]. The feasibility and potential benefits of blocking autophagy in multiple cancers, such as glioblastoma, pancreatic cancer, melanoma, sarcoma, and multiple myeloma, have been demonstrated in clinical trials, including improving tumor control and prolonging median survival [33]. However, excess autophagy induced by 6-shogaol leads to cellular suicide of A549 cells [34]. To our knowledge, few studies have assessed the type of autophagy according to the intensity of the stimulus. A previous study showed that cytotoxins with weak toxins or short exposure induced protective autophagy [35]. In contrast, the same toxic chemicals at high concentrations or prolonged exposure activated death-promoting autophagy in the same cells [35]. In the present study, the transition point of inducing cytoprotective or lethal autophagy through RSV treatment was determined to be 55 μM when autophagy was inhibited by 3-MA. Treatment with RSV lower than 55 μM suppressed cell viability, initiating the activation of protective autophagy. Conversely, incubation with RSV higher than 55 μM elevated cell viability, stimulating lethal autophagy in A549 cells. Determining the concentration threshold is constructive for selecting therapeutic RSV concentrations because we aimed to induce cytoprotective autophagy in normal cells and lethal autophagy in cancer cells with the same RSV content. Our results explored the autophagy type based on the treatment time with different RSV treatment concentrations. It is worth probing the effect of RSV treating time and concentration on autophagy type with response surface method. A similar approach could also be utilized to judge autophagy type activated by other stimuli in other cells. The method may be applied to experimental animals, which requires further research.

Based on the above results, inducing different types of autophagy by different concentrations of RSV, we explored the crosstalk between autophagy and apoptosis in distinguishing autophagy type. The relationship between autophagy and apoptosis is complex. These two processes share common upstream triggers, and autophagy-related proteins and apoptotic proteins (BCL-2 and caspase families) function in both autophagy and apoptosis [9,12]. The autophagy–apoptosis crosstalk has broad implications on the fate of cancer cells [8], which is of significant interest to researchers. It was reported that the inhibition of autophagy (induced by RSV and geftinib + RSV, respectively) significantly promoted apoptosis, indicating autophagy has an antagonistic effect on apoptosis [10,11]. In contrast, blocking apoptosis did not affect autophagy induced by combined geftinib + RSV [11]. However, these studies did not differentiate the dual function of autophagy. Based on the RSV concentration threshold in the present study, we induced cytoprotective autophagy and lethal autophagy by 30 and 80 μM RSV in A549 cells, respectively, and the relationship between autophagy and apoptosis using autophagy inhibitor 3-MA and apoptosis inhibitor Z-VAD-FMK was elucidated. The results indicate that inhibiting protective and lethal autophagy decreased the apoptosis rate. Conversely, apoptosis impairment suppressed death-promoting autophagy but had no impact on survival-promoting autophagy. This might result from the synergistic action between autophagy and apoptosis. Since cytoprotective autophagy occurred prior to apoptosis [36], apoptosis did not affect cytoprotective autophagy. The relationship between lethal autophagy and apoptosis induced by 2,2′,4,4′-tetrabromodiphenyl ether and andrographolide analogue in neuroendocrine pheochromocytoma PC12 cells and human leukemic U937 cells, respectively, was similar to our findings: autophagy inhibition disrupted apoptosis, and apoptosis impairment facilitates autophagy [37,38]. In addition, autophagy induced by fucoxanthin in SGC-7901 cells elevated apoptosis but was unaffected by apoptosis [36]. The different conclusions on the interactions between autophagy and apoptosis could be explained by different types or intensities of stimuli and diverse types or statuses of cells [39,40,41]. In the present study, autophagy type was first determined when investigating the relationship between RSV-induced autophagy and apoptosis in A549 cells, which laid a foundation for further elucidating the correlation between autophagy and apoptosis. Yet, the potential molecular mechanisms required further exploration.

Considering the critical roles of autophagy and apoptosis in cancer cell death and the intricate relationship between them, we further investigated the molecular mechanism of RSV-induced autophagy and apoptosis. We found that RSV targeted the NGFR-AMPK-mTOR signaling pathway to modulate autophagy and apoptosis in A549 cells. RSV activated NGFR by increasing its mRNA expression and prolonging the lifespan of mRNA and protein. RSV reversed the inhibition of autophagy and apoptosis caused by NGFR knockdown, as detected by flow cytometry and qRT-PCR. NGFR modulates autophagy and apoptosis in many cells. For example, rapamycin transactivated NGFR promoter by inhibiting mTOR and activating EGR1 nuclear translocation, leading to upregulated NGFR expression in HK-2 cells. The increased NGFR induced autophagy to mitigate renal tubular damage caused by proteinuria [42]. NGFR enhanced chemosensitivity of colorectal cancer cells to 5-fluorouracil and increased 5-fluorouracil-induced apoptosis and autophagy [15]. Different cells responded differently to NGFR based on cell type and cell differentiation status. For example, NGFR inhibited p73 mRNA and p73-mediated apoptosis in H1299 cells by binding to the p73 central DNA-binding domain [43]. A previous study reported no NGFR expression was detected in lung cancer, but our results did not agree with this conclusion [44]. We further investigated the potential downstream pathway of NGFR in RSV-induced autophagy and apoptosis in A549 cells. It was reported that NGFR could activate AMPK and mTOR via phosphorylation in Schwann cells [19]. As energy sensors, AMPK and mTOR are closely related to autophagy and apoptosis of tumor cells. For example, activating AMPK-mTOR-ULK1 reinforced autophagy, apoptosis, and cytotoxicity in human bladder cancer induced by10-hydroxycamptothecin [45]. The AMPK-mTOR axis took part in NSCLC treatment by inhibiting cell proliferation, migration, invasion, and glycolysis and activating oxidative stress, autophagy, and apoptosis [46,47,48,49]. In the present study, RSV activated AMPK and mTOR by phosphorylation. Knockdown of NGFR suppressed p-AMPK/AMPK expression and elevated p-mTOR/mTOR expression in RSV-treated A549 cells, suggesting RSV activated AMPK by targeting NGFR. Compared to the RSV treatment group, the ratio of p-mTOR to mTOR increased after Cpd. C treatment. Furthermore, Cpd. C co-treatment impaired autophagy and apoptosis induced by RSV, suggesting AMPK plays a crucial role in mTOR activation, as well as autophagy and apoptosis induction. Overall, these results indicate RSV-induced autophagy and apoptosis through the NGFR-AMPK-mTOR pathway regulation. To the best of our knowledge, this work is the first report to clarify the positive effects of the NGFR-AMPK-mTOR pathway on RSV-induced autophagy and apoptosis in A549 cells. In further studies, we plan to explore the NGFR-AMPK-mTOR pathway and the crosstalk between autophagy and apoptosis in vivo.

The present study shows a new method that can be used to evaluate the role of autophagy induced by different concentrations of RSV. The relationship between RSV-induced apoptosis and different types of autophagy is here discussed on this basis. Furthermore, the NGFR-AMPK-mTOR signaling pathway was proved to exert effects in RSV-induced autophagy and apoptosis in A549 cells. However, the present study also has limitations. For example, the molecular mechanisms underlying the relationship between apoptosis and different types of autophagy have not been fully elucidated. Moreover, we only inhibited NGFR or AMPK with siRNA or inhibitor and did not perform overexpression experiments. The lack of any in vivo study also limits the generalization and application of the results. We will focus on these limitations in our future work.

## 5. Conclusions

In summary, the role of RSV-induced autophagy is related to RSV concentration in A549 cells. RSV concentrations lower than 55 μM activated protective autophagy, while RSV concentrations higher than 55 μM stimulated lethal autophagy. The crosstalk between RSV-induced autophagy and apoptosis is complicated. Autophagy increased the apoptosis rate, and apoptosis elevated death-promoting autophagy. In contrast, survival-promoting autophagy was not influenced by apoptosis. RSV induced autophagy and apoptosis by activating the NGFR-AMPK-mTOR pathway in A549 cells. Specifically, RSV increased NGFR protein by increasing mRNA expression and prolonging the lifespan of mRNA and protein. The phosphorylation of AMPK and mTOR were elevated and suppressed after RSV treatment, respectively. RSV could reverse the attenuation of autophagy and apoptosis after NGFR or AMPK was inhibited. These findings provide a foundation for the development of RSV as a new drug and targeted therapy for NSCLC. Future studies will focus on the verification of the mechanisms in vivo.

## Figures and Tables

**Table 1:** Sequences of PCR primers.

Genes	Forward Primers	Reverse Primers
ACTB	5′-CCTGGGCATGGAGTCCTGTG-3′	5′-TCTTCATTGTGCTGGGTGCC-3′
BECLIN 1	5′-GAGGTTGAGAAAGGCGAGACA-3′	5′-GAGGACACCCAAGCAAGACC-3′
LC3B	5′-TTCAGGTTCACAAAACCCGC-3′	5′-TCTCACACAGCCCGTTTACC-3′
BAX	5′-CGGGTTGTCGCCCTTTTCTA-3′	5′-GGAGACAGGGACATCAGTCG-3′
BCL-2	5′-GGGCCGTACAGTTCCACAAA-3′	5′-CTTTGAGTTCGGTGGGGTCA-3′
NGFR AMPK	5′-CTCCAGAACAAGACCTCATAGC-3′ 5′-TCAGGGACTGCTACTCCACA-3′	5′-GATGGAGCAATAGACAGGGATG-3′ 5′-TCCAGGTCTTGGAGTTAGGTCA-3′

## Data Availability

The data presented in this study are available in this article and Supplementary Material.

## Supplementary Materials

The following supporting information can be downloaded at: https://www.mdpi.com/article/10.3390/nu14122413/s1, Figure S1: NGFR knockdown inhibited autophagy and apoptosis in A549 cells; Figure S2: Inhibition of AMPK suppresses resveratrol (RSV)-mediated autophagy and apoptosis.
