# Supplementary material for: Resveratrol Induces Autophagy and Apoptosis in Non-Small-Cell Lung Cancer Cells by Activating the NGFR-AMPK-mTOR Pathway
Source: Nutrients. 2022 Jun 10;14(12):2413. doi: 10.3390/nu14122413 (PMC9228598; doi:10.3390/nu14122413)
Supplement: Supplementary file 1 [file nutrients-14-02413-s001.zip › nutrients-1732342-supplementary.pdf]

nutrients

MDPI

Article

Resveratrol induces autophagy and apoptosis in non-small-cell lung cancer cells by activating the NGFR-AMPK-mTOR pathway

Jiaqiao Li [1], Yameng Fan [1], Yan Zhang [1], Yamei Liu [1], Yan Yu [1]* and Mao Ma [2]*

[1] School of Public Health, Xi'an Jiaotong University, Xi'an, Shaanxi, 710061, P. R. China; yuyan@mail.xjtu.edu.cn (Y.Y.), ljq0321@stu.xjtu.edu.cn (J.L.).
[2] Physical Examination Department, The First Affiliated Hospital of Xi'an Jiaotong University, Xi'an, Shaanxi, 710061, P.R. China; mamao2007@163.com (M.M.).
* Correspondence: yuyan@mail.xjtu.edu.cn (Y.Y.), Tel.: +86 029 82655111 (Y.Y.); mm85323636@xjtufh.edu.cn (M.M.), Tel.: +86 029 85323636 (M.M.)

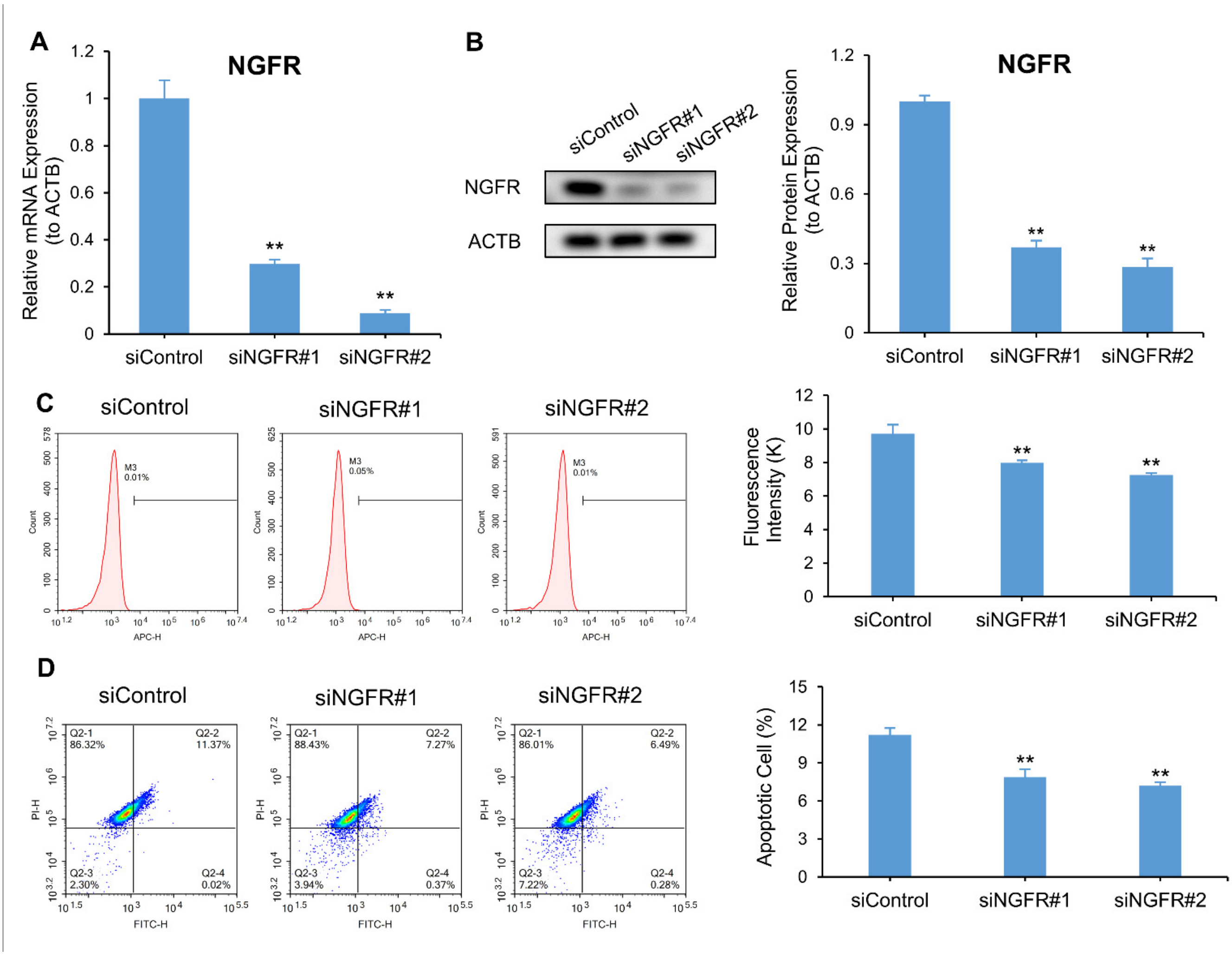

Figure S1. NGFR knockdown inhibited autophagy and apoptosis in A549 cells. Cells were transfected with siControl, siNGFR#1, or siNGFR#2. (A) Transcriptional expression of NGFR as determined by qRT-PCR. ACTB was used as an internal control; (B) Protein expression of NGFR as determined by Western blot. ACTB was used as an internal control; (C) AO (1 μg/mL) staining of autophagic vesicles. Fluorescence intensity was determined by flow cytometry (APC); (D)

TUNEL-FITC/PI staining was used to detect the percentage of apoptotic cells. The data are presented as mean ± SD from three independent experiments. * $p < 0.05$ and ** $p < 0.01$ compared with the control group.

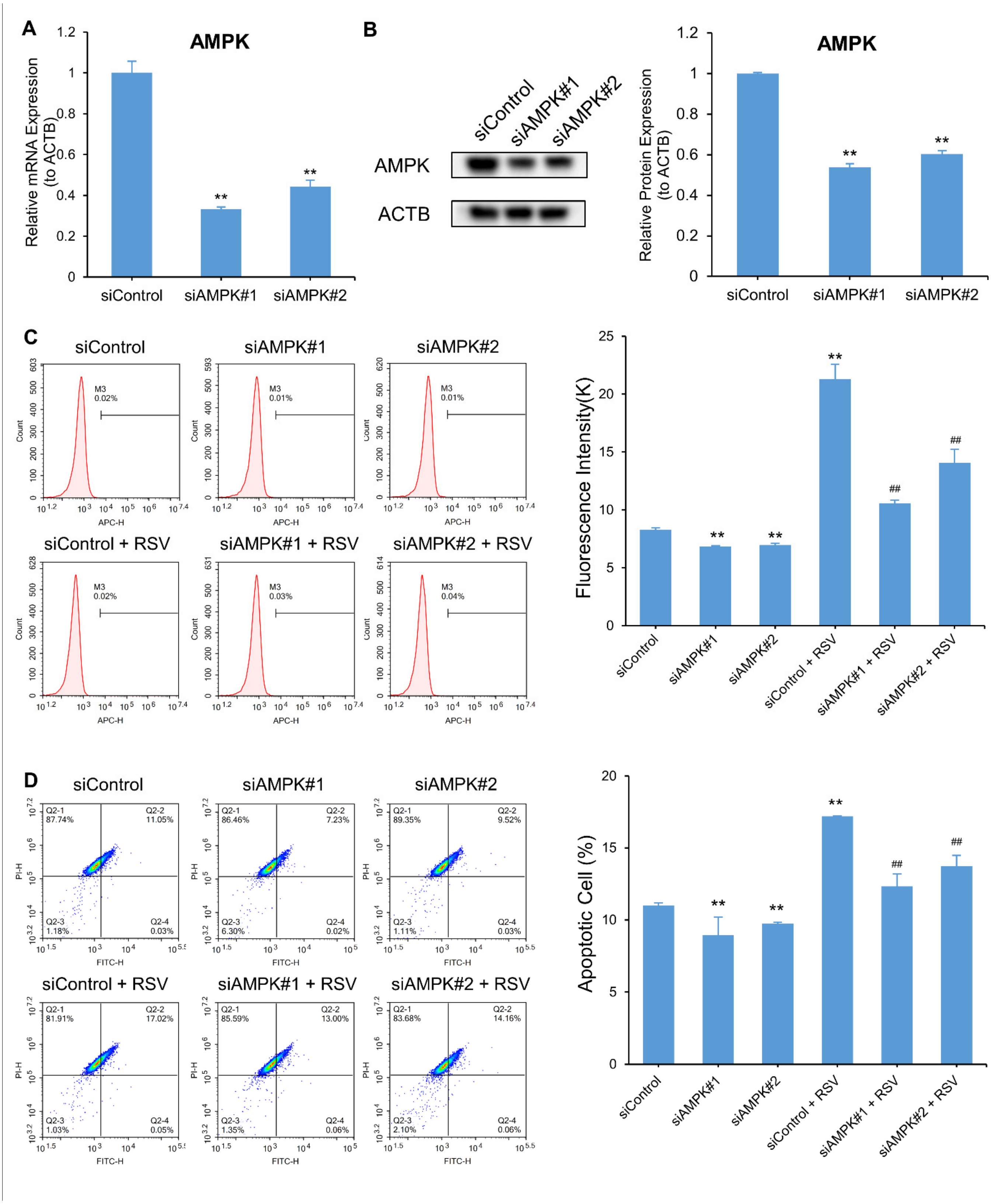

Figure S2. Inhibition of AMPK suppresses resveratrol (RSV)-mediated autophagy and apoptosis. Cells were transfected with siControl, siAMPK#1, or siAMPK#2, in the presence or absence of 80 μM RSV for 48 h. (A) Transcriptional expression of AMPK as determined by qRT-PCR. ACTB was used as an internal control; (B) Protein expression of AMPK as determined by Western blot. ACTB was used as an internal control; (C) AO (1 μg/mL) staining of autophagic vesicles. Fluorescence intensity was determined by flow cytometry (APC); (D) TUNEL-FITC/PI staining was used to detect the percentage of apoptotic cells. The data are presented as mean ± SD from three independent experiments. * $p < 0.05$ and ** $p < 0.01$ compared with the control group. # $p < 0.05$ and ## $p < 0.01$ compared with RSV group.
